# Erratum: Development and Implementation of a Geriatric Oncology Interdisciplinary Case–Based Educational Intervention for Cancer Care Providers

**DOI:** 10.1200/GO-25-00029

**Published:** 2025-02-20

**Authors:** Haydee C. Verduzco-Aguirre, Carolina Gomez-Moreno, Ana P. Navarrete-Reyes, Gretell Henriquez-Santos, Javier Monroy Chargoy, Abigail Mateos-Soria, Juan José Sánchez Hernández, Alicia Castelo-Loureiro, Liz Hamui-Sutton, Melchor Sánchez-Mendiola, Enrique Soto-Perez-de-Celis

The article by Verduzco-Aguirre et al entitled “Development and Implementation of a Geriatric Oncology Interdisciplinary Case–Based Educational Intervention for Cancer Care Providers” (*JCO Global Oncology* 10.1200/GO-24-00258) was published online October 17, 2024, with errors.

Acknowledgments were omitted from the article.

Support

“We thank the Mexican Society of Oncology (Sociedad Mexicana de Oncología) and the National College of Geriatric Medicine (Colegio Nacional de Medicina Geriátrica) for their contributions to the dissemination of this project and their endorsement of the educational intervention.”

This has been corrected as of February 20, 2025. The authors apologize for the errors.

